# Supplementary material for: Surgical and Oncological Outcomes of En-Bloc Resection for Malignancies Invading the Thoracic Spine
Source: J Clin Med. 2022 Dec 20;12(1):31. doi: 10.3390/jcm12010031 (PMC9820992; doi:10.3390/jcm12010031)
Supplement: Supplementary file 1 [file jcm-12-00031-s001.zip › Supplementary Table S1.pdf]

Supplementary Table S1. Outcomes of the sample based on complications. The analysis of data showed that the variables related to a higher probability of developing complications are "age", presence of "pneumological comorbidities" and hospital stay. In the multivariate logistic model only age and hospital stay were confirmed as a statistically significant factor for the development of complications.

	Univariate analysis			Logistic model for complications		
	**No complications (N: 31)**	**Complications (N: 41)**	**P value**	**Odds Ratio**	**Conf. Interval**	**P value**
**Age, median – year (Mean±SD)**	41±18.2	52±16.3	**0.0061**	1.03	1.00-1.06	**0.04**
**Gender, male – n (%)**	17 (55%)	28 (68%)	0.24			
**Cardiological comorbitities – n (%)**	6(19%)	10(24%)	0.61			
**Pneumological comorbitities – n (%)**	1(3.2%)	9(21%)	**0.036**	5.02	0.56-44.72	0.14
**Metabolic comorbitities – n (%)**	4(12%)	9(21%)	0.37			
**Pre-Operative ASIA score**			0.22			
- **A – n(%)**	1(3.2%)	1(2.4%)				
- **B – n(%)**	0(0%)	4(9.7%)				
- **C – n(%)**	6(19%)	6(14%)				
- **D – n(%)**	3(9.6%)	9(21%)				
- **E – n(%)**	21(67%)	21(51%)				
**Pre-operative chemotherapy – n (%)**	11(35%)	16(39%)	0.75			
**Pre-operative Radiotherapy – n (%)**	7(22%)	13(31%)	0.39			
**No. of involved vertebrae**			0.55			
- **1 – n (%)**	12(38%)	11(26%)				
- **2 – n (%)**	8(25%)	12(29%)				
- **3 – n (%)**	11(35%)	18(43%)				
**Associated lung resection – n (%)**	10(32%)	17(41%)	0.42			
**Surgical incisions**			0.30			
- **Posterior Incision alone – n (%)**	11(35%)	10(24%)				
- **Posterior incision and thoracotomy – n (%)**	20(64%)	31(75%)				
**Tumors Group**			0.23			
- **1 – n (%)**	20(64%)	20(48%)				
- **2 – n (%)**	4(12%)	12(29%)				
- **3 – n (%)**	7(22%)	9(21%)				
**Surgical time-Hours (Medians IQR)**	520(360-600)	570(480-699)	0.095			
**ICU stay-Days (Medians IQR)**	1(1-4)	2(1-6)	0.083			
**Hospital stay-Days (Medians IQR)**	17(12-20)	21(14-27)	**0.030**	1.07	1.00-1.14	**0.036**
